# Effects of Physical Modification Methods on Physicochemical, Structural and Functional Characteristics of Insoluble Dietary Fiber from Okara

**DOI:** 10.3390/foods15142456

**Published:** 2026-07-10

**Authors:** Xuyao Wei, Huanyu Zheng

**Affiliations:** 1 College of Food Science, Northeast Agricultural University, Harbin 150030, China; weixy2000172026@163.com; 2 Heilongjiang Green Food Science Research Institute, Harbin 150028, China

**Keywords:** okara, IDF, physical modification, bioactivity

## Abstract

Physical treatment can improve the quality and overall characteristics of insoluble dietary fiber (IDF). This study investigated the effects of microjet homogenization treatment (MT), ultra-high-pressure treatment (HP), and ultrasonic treatment (UT) on the compositional profile, microstructure, basic properties, and bioactivity of IDF from okara. The modification processes increased IDF yield (unmodified IDF: 65.55%; MT-IDF: 70.51%; HP-IDF: 75.29%; UT-IDF: 79.09%). The mechanical action disrupted the compact structure, refined the particles, and increased the specific surface area (SSA). Compared with unmodified IDF (0.18 m^2^/g), the SSA values of MT-IDF, HP-IDF and UT-IDF increased by 83.33%, 50.00%, and 72.22%, respectively, thereby improving the overall hydration characteristics and adsorption performance of IDF. UT-IDF exhibited excellent water-holding capacity (8.11 g/g), yet its thermal stability ((mass loss: unmodified IDF (36.50%), MT-IDF (23.59%), HP-IDF (26.11%), and UT-IDF (33.74%)) and rheological properties (shear rate range of 35–40 s^−1^: unmodified IDF (1.77 Pa·s), MT-IDF (15.86 Pa·s), HP-IDF (21.11 Pa·s), UT-IDF (5.36 Pa·s)) were relatively inferior to those of MT-IDF and HP-IDF. Notably, MT-IDF exhibited superior modification effects, including a loose, porous microstructure, enhanced adsorption performance, and favorable prebiotic potential (*Lactobacillus acidophilus* 36h-OD_600_: 0.594; *Bifidobacterium longum* 36h-OD_600_: 0.509). Among the three physical modification methods, MT offers significant advantages in enhancing the quality of IDF from okara, improving its processing suitability, and facilitating its high-value utilization. However, this study still has certain limitations: the evaluation of the correlation between fiber digestion rate and probiotic potential was based only on in vitro models and has not been systematically evaluated using real food matrices.

## 1. Introduction

Okara is a major by-product generated during the processing of soybean products, including tofu and soy milk, and accounts for approximately 15% to 20% of the dry weight of soybeans [1]. With the rapid development of the soybean deep-processing industry, large quantities of okara are discarded because of its high moisture content, thereby worsening environmental pollution. In both wet and dried forms, okara can be incorporated into diverse food formulations as a food additive or binder to improve the nutritional and functional attributes of final products [2]. However, okara possesses multiple inherent drawbacks that limit its use in food processing: it is highly perishable and exhibits low digestibility, while abundant endogenous aldehydes generate undesirable beany off-odors; in addition, its high fiber content results in a gritty texture, thereby restricting its large-scale utilization in the food industry [3]. Okara is rich in dietary fiber (42.4–58.1 g/100 g), protein (15.2–33.4 g/100 g), total phenolics (122.57 mg gallic acid equivalents/100 g), calcium (260–428 mg/100 g), and various biologically active substances [2,4]. Dietary fiber (DF) in okara is divided into two categories: soluble dietary fiber (SDF) and insoluble dietary fiber (IDF), of which IDF accounts for 70% of the total dietary fiber [5]. Adequate intake of IDF plays a vital role in maintaining health. In the context of green and sustainable food development, the efficient recovery and value-added conversion of food manufacturing by-products have attracted widespread attention.

DF is associated with numerous health benefits and is commonly classified as SDF or IDF according to its solubility. IDF is mainly composed of non-starch polysaccharides, including cellulose and hemicellulose, as well as lignin. Owing to its inherent metabolic inertness and molecular structure rich in hydroxyl, carboxyl, and other active moieties, IDF can effectively adsorb glucose, bile salts, and cholesterol [6,7]. Long-term intake of IDF can regulate the gut microbiota, prevent constipation, and lower the risk of metabolic disorders. SDF is more readily applicable than IDF in liquid, semi-solid, and solid food systems [8]. Furthermore, SDF offers distinct advantages in terms of emulsification, stability, and texture modification. However, SDF generally constitutes only a small proportion of natural raw materials and is difficult to extract, whereas IDF accounts for a higher proportion of total dietary fiber. Natural IDF generally has relatively large particle sizes and a dense network structure, making it difficult for its internal active groups to be fully exposed, which in turn adversely affects its physicochemical properties and functional activity [9]. Therefore, using appropriate modification methods to tailor the chemical structure of IDF-derived polymers can effectively enhance their functional properties, thereby expanding their application prospects in the functional food sector.

The main methods used to modify DF fall into three categories: physical, chemical, and biological methods. Chemical and biological modifications can alter the functional groups of DF and yield modified fibers with excellent polymerization performance and high purity. Nevertheless, these approaches still exhibit notable shortcomings, including chemical residues, lengthy reaction cycles, and limitations in industrialization [10]. Physical modification has the advantages of being environmentally friendly, cost-effective, and easy to operate, making it better suited to the demands of industrial production [11,12]. Microjet homogenization generates high-speed shear, convective collisions, and cavitation effects within a high-pressure chamber, promoting particle refinement and modulating the physicochemical and functional attributes of DF [13]. Li et al. [14] reported that microjet homogenization (100 MPa) reduced the particle size of coconut pomace DF by 85.18% and increased its specific surface area by 164.53%, while also altering its crystallinity and monosaccharide composition. Ultra-high-pressure treatment is a non-thermal modification strategy. This modification process is not restricted by material form, and pressure transmission is independent of material volume or processing duration, thereby shortening processing cycles and broadening its potential commercial applications [15]. Zuo et al. [16] demonstrated that ultra-high-pressure treatment reduced the molecular weight of litchi pomace SDF (PSDF). Compared with untreated SDF, modified PSDF promoted the growth of beneficial bacteria (*Lactobacillus rhamnosus*; *Lactobacillus acidophilus*). This treatment can also significantly improve the aggregation characteristics of DF and its interaction with water, thereby optimizing its physicochemical properties. Ultrasonic treatment can induce intense vibrations in polar molecules, generating cavitation effects that break polysaccharide chemical bonds, thereby improving their surface physicochemical properties and enhancing functional activity [17]. Hussain et al. [18] demonstrated that ultrasonic treatment improved the hydration properties of sea buckthorn pomace DF, while also inducing significant color changes in both IDF and SDF. All three physical modification methods rely on mechanical forces to disrupt the dense aggregation structure of fibers, thereby enhancing their functional properties; they are environmentally friendly and highly suitable for industrial applications. Rather than focusing on process optimization, this study adopted representative modification conditions reported in the literature and systematically compared the differential regulatory patterns of three physical modification methods on the physicochemical properties of okara IDF. This approach avoids the inherent limitations of chemical and biological modification and provides theoretical guidance for the high-value valorization and industrial development of okara IDF.

Safe and environmentally friendly physical modification strategies are an effective means of enhancing the bioavailability of DF. However, there remains a lack of systematic comparisons of the mechanisms by which different physical modification methods regulate the structure–activity relationship of IDF in okara. This study aims to investigate the mechanisms by which different physical treatments modulate the compositional, microstructural, and functional properties of okara IDF, thereby supporting industrial-scale production of okara IDF and the development of functional food formulations.

## 2. Materials and Methods

### 2.1. Materials

Sterile okara was procured from a tofu processing factory (Harbin, China), with a powder moisture content of 6.70%. Heat-stable α-amylase (20 U/mg), neutral protease (50 U/mg), and amyloglucosidase (100 U/mg) were purchased from Shifeng Biotechnology Co., Ltd. (Shanghai, China). Simulated salivary fluid (SSF, containing α-amylase ≥ 3.7 U/mg protein), simulated gastric fluid (SGF, containing pepsin ≥ 2.2 U/mg protein), and simulated intestinal fluid (SIF, containing trypsin ≥ 2.5 U/mg protein) were all procured from Feijing Biotechnology Co., Ltd. (Fuzhou, China). *Lactobacillus acidophilus* (*L. acidophilus*) and *Bifidobacterium longum* (*B. longum*) were purchased from Zhongke Jiayi Biological Engineering Co., Ltd. (Jinan, China). Sodium cholate, furfural, and silver nitrate were purchased from Solarbio Science & Technology Co., Ltd. (Beijing, China). Anhydrous glucose was purchased from Huasheng Reagent Co., Ltd. (Tianjin, China).

### 2.2. Preparation of IDF

**Figure 1:**
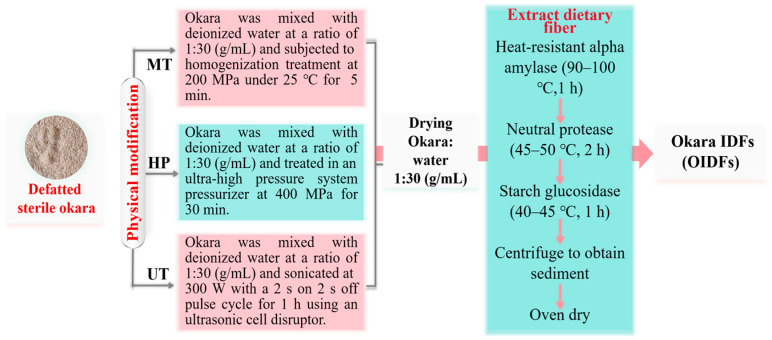
Procedure sketch of the method used to modify okara IDF.

The IDF extraction and modification process from okara is illustrated in Figure 1.

#### 2.2.1. Unmodified IDF

The raw materials were prepared according to the method reported by Tian et al. [19], with minor modifications. Defatted okara powder passed through a 50-mesh sieve was mixed with deionized water at a solid–liquid ratio of 1:30 (g/mL). The pH was adjusted to 4.2 and monitored using a pH meter (FE22-Standard, Mettler Toledo, Zurich, Switzerland). Then, 1% heat-stable α-amylase was added, and the suspension was hydrolyzed at 90–100 °C for 1 h to preliminarily degrade the starch fractions. After the first enzymatic hydrolysis, the suspension was cooled to 45–50 °C, and the pH was adjusted to 7.0 before 2% neutral protease was added. The suspension was incubated for 2 h to fully degrade the protein components in the sample. Following protease hydrolysis, the suspension was further cooled to 40–45 °C, and the pH was readjusted to 4.2. Afterward, 1% amyloglucosidase was added and allowed to react for 1 h to eliminate residual starch impurities. Enzymes were inactivated by boiling for 10 min after each enzymatic step. Once the suspension had cooled to room temperature, it was centrifuged at 5000 rpm for 20 min, and the resulting precipitate was collected. The precipitate was rinsed three times with deionized water and dried in a forced-air oven at 50 °C for 48 h to yield unmodified okara insoluble dietary fiber, denoted CB-IDF.

#### 2.2.2. Microjet Homogenization Treatment

The procedure was performed according to the method of Kang et al. [13]. A total of 50 g of defatted okara powder was thoroughly mixed with 1.5 L of deionized water to form a homogeneous suspension without particle agglomeration. The mixture was treated using a high-pressure water-jet homogenizer (HPW-10, Hilock, Shanghai, China) at 200 MPa for 5 min. After homogenization, the treated sample was dried in a forced-air oven at 50 °C for 48 h to remove residual moisture. Subsequently, the sample was processed in accordance with Section 2.2.1 and labeled MT-IDF.

#### 2.2.3. Ultra-High Pressure Treatment

This procedure was adapted, with slight modifications, from the method described by Ma et al. [20]. A total of 50 g of defatted okara powder was thoroughly mixed with 1.5 L of deionized water to form a homogeneous suspension without particle agglomeration. The mixture was then placed in a pressure vessel (2 L high-performance ultra-high-pressure apparatus, Lidefu Technology Co., Ltd., Shenzhen, China) and treated at 400 MPa for 30 min. The treated sample was dried in a forced-air oven at 50 °C for 48 h to remove residual moisture. The sample was subsequently processed in accordance with Section 2.2.1 and labeled HP-IDF.

#### 2.2.4. Ultrasonic Treatment

The method was performed according to Shi et al. [21]. A total of 50 g of defatted okara powder was thoroughly mixed with 1.5 L of deionized water to form a homogeneous suspension without particle agglomeration. The mixture was then treated using an ultrasonic cell disruptor (JY 92-IIN, Scientz, Ningbo, China) at 300 W for 1 h, with a cycle of 2.0 s on and 2.0 s off. The treated sample was dried in a forced-air oven at 50 °C for 48 h to remove residual moisture. Subsequently, the sample was processed in accordance with Section 2.2.1 and labeled UT-IDF.

### 2.3. Basic Characteristics

#### 2.3.1. IDF Yield and Composition

The IDF content was determined in accordance with AOAC Method 991.43; crude protein content was determined using a fully automated Kjeldahl analyzer (GK-600, Glkrui, Shanghai, China) in accordance with AOAC Method 950.40. Ash content was determined by dry ashing (SX2-4-10N, Yiheng Scientific Instrument Co., Ltd., Shanghai, China) in accordance with AOAC Method 923.03.

#### 2.3.2. Color and Particle Size

A colorimeter (DS-200, Caipu Technology Co., Ltd., Hangzhou, China) was used to measure the color parameters L*, a*, and b* of the IDF samples. The color differences among the various modified samples were quantified.

IDF was dispersed in distilled water at a ratio of 1:10. After thorough mixing, particle size was measured using a laser particle size analyzer (LS-POP(9), Addison, Eurok, TX, USA). The refractive indices were set to 1.537 for IDF and 1.333 for water.

### 2.4. Structural Characterization

#### 2.4.1. Scanning Electron Microscopy (SEM)

The morphology of the IDF was examined using SEM (SU8010, Hitachi, Tokyo, Japan) at 25 °C and an acceleration voltage of 5 kV. The IDF samples were fixed onto a sample holder coated with conductive carbon tape, sputtered with gold, and observed at magnifications of 500×, 2000×, and 5000×.

#### 2.4.2. Fourier-Transform Infrared Spectroscopy (FTIR)

FTIR (Nicolet IS50, Thermo Fisher Scientific, HE, Germany) was used to analyze the functional groups of IDF. The IDF sample was mixed with dried potassium bromide at a mass ratio of 1:100 and ground until no visible granules remained. Measurements were performed at room temperature, with the wavenumber scan range set from 4000 cm^−1^ to 400 cm^−1^.

#### 2.4.3. X-Ray Diffraction (XRD)

The crystalline structures of IDF were characterized and analyzed via X-ray diffraction (SmartLab SE, Rigaku, Tokyo, Japan). The measurement range was 5–60° (2θ), and the scanning rate was 2°/min. The XRD spectra were processed for peak deconvolution using OriginPro software (Version 2026, OriginLab Corporation, MA, USA). Relative crystallinity was calculated by integrating the areas of crystalline and amorphous peaks, and the results were expressed as percentages (%).

#### 2.4.4. Viscosity (VS)

This method was slightly modified from the approach described by Geng et al. [22]. Measurements were carried out using a hybrid rheometer (HAAKE MARS60, Thermo Fisher, BW, Germany). IDF powder was mixed with pure water to prepare an aqueous dispersion with a concentration of 0.2 g/mL. The temperature was set to 20 °C and the shear time was set to 60 s. The test was conducted at a shear rate of 0 to 200 s^−1^.

#### 2.4.5. X-Ray Photoelectron Spectroscopy (XPS)

The XPS instrument (Thermo K-Alpha, Thermo Fisher Scientific, MA, USA) was used to analyze the elemental composition of the IDF powder. The excitation source was Al kα radiation, and the vacuum level in the analysis chamber was 5 × 10^−9^ mbar. The analysis energy was set at 50 eV, with a step size of 0.1 eV. Peak fitting was performed using Avantage software (Version 5.9921, Thermo Fisher Scientific, MA, USA).

#### 2.4.6. Thermogravimetric Analysis (TG)

The analysis was carried out using a thermogravimetric analyzer (STA2500, Netzsch, BY, Germany) under a nitrogen atmosphere. A 15 mg portion of the okara IDF was accurately weighed and placed in an aluminum crucible; the IDF was then heated from 25 °C to 600 °C at a heating rate of 10 °C/min.

### 2.5. Physical and Chemical Properties

#### 2.5.1. Water-Holding Capacity (WHC)

A 0.2 g sample was accurately weighed, mixed with 20 mL of deionized water, and shaken thoroughly for 2 h. The mixture was kept at ambient temperature for 18 h and then centrifuged at 4000 rpm for 120 min. The supernatant was removed, residual moisture on the container walls was wiped off with filter paper, and the precipitate was weighed.

(1) $$WHC(g/g)=\frac{M_{\;b}-{\;M}_{a}}{M_{a}}$$

where *M_b_* represents the wet weight of the sample after centrifugation, and *M_a_* represents the dry weight of the sample.

#### 2.5.2. Swelling Capacity (SC)

A 0.2 g sample of okara IDF was accurately weighed, mixed thoroughly with 20 mL of deionized water, and left to stand at ambient temperature for 24 h [23]. The initial volume of the sample system and the volume after swelling equilibrium were recorded.

(2) $$SC(mL/g)=\frac{V_{b}-{\;V}_{a}}{M_{a}}$$

where *V_b_* represents the volume of the sample after swelling equilibrium was reached, *V_a_* represents the initial volume of the sample, and *M_a_* represents the dry weight of the sample.

#### 2.5.3. Glucose Adsorption Capacity (GAC)

Minor modifications were made to the established method proposed by Yang et al. [24]. A 0.2 g sample of IDF powder was fully mixed with 20 mL glucose solution at concentrations of 10 mmol/L and 50 mmol/L, respectively. The mixed suspensions were incubated at 37 °C for 6 h before centrifugation at 8000 rpm for 20 min, and the resulting supernatants were retained for subsequent colorimetric reaction. A 0.5 mL aliquot of each supernatant was mixed with DNS reagent, and the mixture was heated in a boiling water bath for 5 min for color development. After cooling, the reacted solutions were diluted to an appropriate concentration, and their absorbance values were determined at 540 nm (T6, PERSEE, Beijing, China).

(3) $$GAC(mmoL/g)=\frac{X_{b}-X_{a}}{M_{a}}$$

where *X_b_* is the initial glucose content, *X_a_* is the glucose content after adsorption, and *M_a_* is the dry weight of the sample.

#### 2.5.4. Sodium Cholate Adsorption Capacity (SCAC)

Minor adjustments were made to the protocol described by Xie et al. [25]. A 0.2 g sample of IDF powder was fully blended with 20 mL of sodium cholate solution (0.75 mg/mL). The obtained mixtures were separately adjusted to pH 2.0 ± 0.1 and pH 7.0 ± 0.1 using a pH meter (PHS-3C, INESA Scientific Instrument Co., Ltd., Shanghai, China), and were then incubated for 2 h and 3 h, respectively. Upon completion of the reaction, the mixtures were centrifuged at 5000 rpm for 15 min, and the residual sodium cholate content in the collected supernatants was quantified using the furfural colorimetric method.

(4) $$SCAC(mg/g)=\frac{W_{b}-W_{a}}{M_{a}}$$

where *W_b_* is the initial sodium cholate content, *W_a_* is the sodium cholate content after adsorption (mg), and *M_a_* is the dry weight of the sample.

#### 2.5.5. Cation Exchange Capacity (CEC)

A 0.5 g sample was thoroughly blended with 30 mL of hydrochloric acid solution (0.1 M) and left to acidify for 24 h. Upon completion of acidification, the mixture was filtered under vacuum; the residue was then repeatedly washed with pure water until no Cl^−^ was observed in the filtrate using a 10% silver nitrate solution. The washed residue was vacuum-dried at 40 °C, after which 0.1 g of the dried sample was weighed and added to 50 mL of sodium chloride solution (5%). A blank control was prepared using distilled water. Titration was conducted using 0.1 M NaOH, with phenolphthalein as the indicator [26,27].

(5) $$CEC(mmoL/g)=0.1\times \frac{V_{1}-V_{0}}{M_{a}}$$

where *V*_1_ is the volume of NaOH consumed in the titration of the sample, *V*_0_ is the volume of NaOH consumed in the blank titration, and *M_a_* is the dry weight of the sample.

### 2.6. Biological Activity

#### 2.6.1. In Vitro Digestion Rate Determination

In this work, the in vitro digestion method previously reported by Yu et al. [28] was adopted with minor modifications. Gastric and intestinal digestion were simulated independently, and the detailed experimental procedures are presented below.

(1) Simulated oral pretreatment solution

A volume of 20 mL of sample solution at a concentration of 25 mg/mL was transferred into a 50 mL sterile test tube. A total of 16 mL of SSF, 100 μL of 0.3 mol/L CaCl_2_ solution, and 3.9 mL of deionized water were sequentially added to the sample solution. After thorough mixing, the mixture was incubated in a shaker (SHA-B, Lichen Instrument Technology Co., Ltd., Shanghai, China) at 37 °C and 180 rpm for 5 min to prepare the oral digestive fluid.

(2) Simulated gastric digestion

An aliquot of 10.0 mL of the prepared oral digestive fluid was mixed with 9.1 mL of SGF, 10 μL of 0.3 mol/L CaCl_2_ solution, and 0.695 mL of deionized water. The pH of the homogeneous mixture was adjusted to 3.0 with 1 M HCl. The resulting mixture was incubated in a shaking incubator (SHA-B, Lichen Instrument Technology Co., Ltd., Shanghai, China) at 37 °C and 180 rpm for 2 h to obtain the gastric digestive solution.

(3) Simulated intestinal digestion

A volume of 20.0 mL of oral digestive fluid was supplemented with 16 mL of SIF, 40 μL of 0.3 mol/L CaCl_2_ solution, and 3.81 mL of deionized water. After complete homogenization, the pH of the solution was adjusted to 7.0 using 1 M NaOH solution. The mixture was incubated in a shaking incubator (SHA-B, Lichen Instrument Technology Co., Ltd., Shanghai, China) at 37 °C and 180 rpm for 2 h to obtain the intestinal digestive fluid.

The obtained gastric and intestinal digestive solutions were individually blended with 15% (*w*/*v*) trichloroacetic acid solution at a volume ratio of 1:1. The mixed solutions were allowed to stand for 5 min before centrifugation at 4000 rpm for 10 min. The content of hydrolyzed peptides in the supernatant was determined by measuring the absorbance at 280 nm (T6, PERSEE, Beijing, China). The relative digestion rate was finally expressed as ΔOD_280nm_/h.

#### 2.6.2. In Vitro Probiotic Activity Determination

Bacterial suspensions for the two probiotic strains were prepared according to Li et al. [29], with slight modifications. Glucose, inulin, CB-IDF, MT-IDF, HP-IDF, and UT-IDF were separately added at a concentration of 2% (*w*/*w*) to carbon-source-free medium, while a carbon-source-free culture served as the blank control. All liquid culture media required for the experiment were autoclaved at 121 °C for 20 min, cooled to ambient temperature, inoculated with the prepared bacterial suspensions at 2% (*v*/*v*), and cultured in an anaerobic incubator at 37 °C.

(1) Growth curve determination: At 0, 4, 8, 12, 16, 20, 24, 28, 32, and 36 h, 1 mL of the fermented bacterial suspension was transferred to sterile test tubes, followed by centrifugation at 2000 rpm for 2 min, and the supernatant was retained. The OD at 600 nm was measured using a 96-well plate, and growth curves were plotted.

(2) pH determination: The pH of the supernatant from step (1) above was measured.

### 2.7. Data Analysis

SPSS 27 was used for statistical analysis, and differences among means were evaluated using Duncan’s test (*p* < 0.05). All data were obtained from three replicates and expressed as mean ± standard deviation. The figures were plotted using OriginPro 2026.

## 3. Results

### 3.1. Yield and Compositional Analysis of IDF

**Figure 2:**
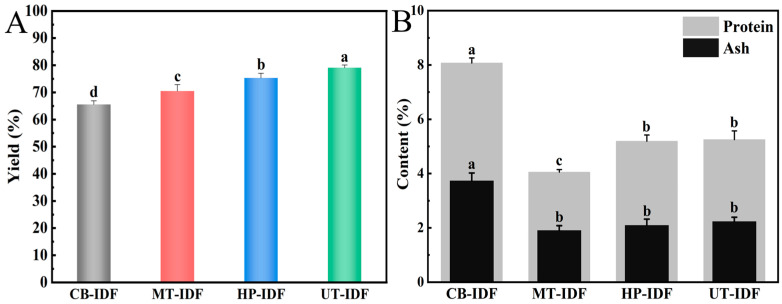
Yield (**A**) and composition (**B**) of IDF samples subjected to different modification methods. CB-IDF: unmodified IDF; MT-IDF: IDF modified by microjet homogenization; HP-IDF: IDF modified by ultra-high pressure; UT-IDF: IDF modified by ultrasound. Values marked with different letters are significantly different (*p* < 0.05).

The effects of different physical modification methods on the extraction yield, protein content, and ash content of IDF from okara are shown in Figure 2. Compared with CB-IDF, the extraction yields of MT-IDF, HP-IDF, and UT-IDF were all significantly higher (*p* < 0.05), with the increases following the order UT-IDF > HP-IDF > MT-IDF (Figure 2A). Ultrasonic treatment exerts an intense impact on DF particles, causing the breakdown of cell wall and membrane structures, thereby allowing the encapsulated IDF to be released more fully; this may explain why UT-IDF had the highest yield [30]. As presented in Figure 2B, the three modification methods reduced the protein and ash contents of IDF to levels markedly lower than those in CB-IDF, which had the highest protein and ash contents. This difference was likely because DF and bound protein particles were embedded within the fiber structure and aggregated into a cohesive mass [31]. MT-IDF exhibited the lowest impurity content, indicating its superior purity. Physical modifications achieved both improved IDF extraction rates and enhanced purity through different mechanisms of action.

### 3.2. Color and Particle Size Difference Analysis

Food color affects both processability and the product’s visual appeal and consumer acceptance. As presented in Table 1, MT-IDF had the highest lightness value, whereas CB-IDF had the lowest. The a* values of MT-IDF, UT-IDF, and HP-IDF were all significantly higher than those of CB-IDF (*p* < 0.05). It is speculated that the resulting reddish tone may make these samples more suitable for meat products, such as sausages and hamburgers, as well as strawberry jam products [24]. The b* value of modified UT-IDF increased markedly, while there was no significant difference between MT-IDF and HP-IDF (*p* > 0.05). This may be due to an increase in small-molecule protein degradation products during ultrasonic treatment. The differences in color among the various modified IDF samples may be related to changes in the content of soluble pigments and proteins within the system [23].

As shown in Table 1, CB-IDF exhibited the widest particle size dispersion and the largest average particle size. The order of particle refinement for the modified IDF was MT-IDF > UT-IDF > HP-IDF. This is related to the fiber-breaking capacity of the different modification methods; the high-speed shear of microjets and the cavitation shock waves of ultrasound can directly sever long fiber chains, forming finer particles and thereby altering the fiber structure [11]. Particle refinement increases the specific surface area of the IDF; research indicates that this enhances the hydration capacity and adsorption properties of IDF [32,33].

### 3.3. Structural Characterization

#### 3.3.1. Scanning Electron Microscopy (SEM)

**Figure 3:**
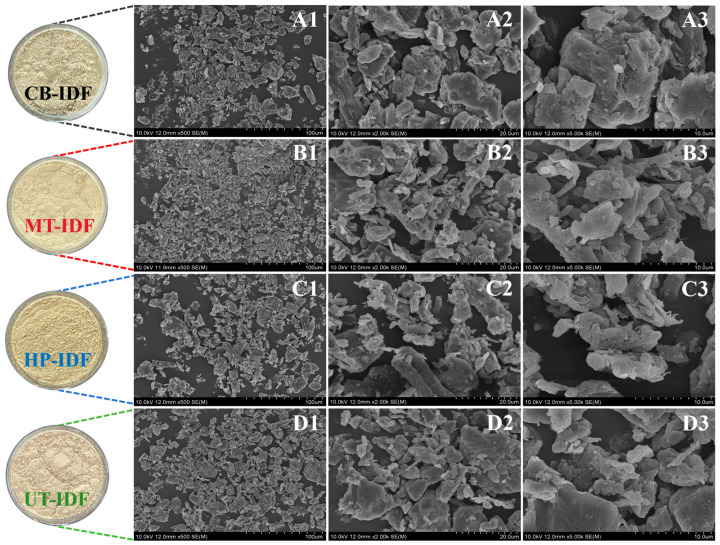
SEM images of IDF samples subjected to different modification methods. CB-IDF: (**A1**–**A3**) (500×, 2000×, 5000×); MT-IDF: (**B1**–**B3**) (500×, 2000×, 5000×); HP-IDF: (**C1**–**C3**) (500×, 2000×, 5000×); UT-IDF: (**D1**–**D3**) (500×, 2000×, 5000×).

SEM is an effective method for revealing the structural characteristics of fiber surfaces. As shown in Figure 3 (magnifications of 500×, 2000×, and 5000×), the surfaces of the modified IDF samples exhibited uneven thickness, a multi-lamellar structure, and distinct structural fractures. The surface of CB-IDF at 5000× magnification showed dense packing accompanied by particle agglomeration, possibly due to impurities such as proteins and ash [34]. MT-IDF exhibited the greatest degree of particle refinement. Strong shear forces disrupted the fiber cross-linked structure, reduced the degree of polymerization, formed a loose, high-void-fraction structure, and promoted the full exposure of active groups, thereby significantly enhancing its adsorption capacity [35,36]. The microstructural characteristics of MT-IDF were in line with the findings in Table 1, which showed the smallest particle size and the largest SSA. At 5000× magnification, the dispersibility of HP-IDF particles was weaker than that of MT-IDF and UT-IDF, but its wrinkled surface may facilitate water penetration and binding. Similar results were obtained in the study by Yang et al. [37]. All three modification methods disrupted the internal structure of okara IDF to varying degrees, contributing to the further optimization of its functional properties.

#### 3.3.2. Fourier-Transform Infrared Spectroscopy (FTIR)

**Figure 4:**
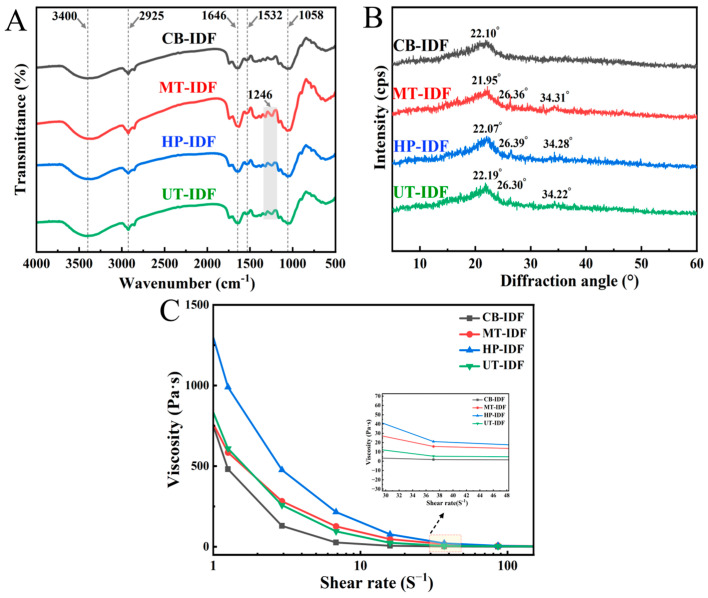
FT-IR (**A**); XRD (**B**); viscosity (**C**) profiles of IDF samples subjected to different modification methods.

As shown in Figure 4A, the three physical modification approaches did not change the primary chemical structure of IDF; differences were observed only in the intensity of the absorption peaks. All four sample groups exhibited stretching vibration absorption peaks near 3400 cm^−1^, 2925 cm^−1^, 1646 cm^−1^, 1532 cm^−1^, and 1058 cm^−1^, corresponding to OH, C-H, C=O, COO^−^, and C-O-C vibrations, respectively [38]. At 1632 cm^−1^, the absorption peak intensities of MT-IDF, HP-IDF, and UT-IDF were stronger than that of CB-IDF, indicating an increase in uronic acid content. The presence of uronic acid enhances the adsorption performance of IDF [39]. Compared with CB-IDF, the absorption peak at 1246 cm^−1^ was more pronounced in the modified IDF; this phenomenon could be explained by the formation of some SDF during the modification process, which was subsequently removed during the washing step, resulting in a slight change in the infrared spectral absorption peak [40]. In summary, the physical modification methods did not disrupt the basic backbone of the polysaccharide, but instead achieved structural optimization by altering intermolecular interactions and the state of aggregation.

#### 3.3.3. X-Ray Diffraction (XRD)

The XRD spectra showed the crystalline structures of the samples before and after modification (Figure 4B). The four groups of IDF samples exhibited a main diffraction peak at approximately 22°, indicating the typical crystalline structure of cellulose I [41]. MT-IDF, HP-IDF, and UT-IDF showed enhanced signals at two secondary diffraction peaks, at 26.30°and 34.30°. Compared with CB-IDF, which had a crystallinity of 6.03%, all three modification methods significantly increased the crystallinity of IDF: MT-IDF (11.21%) > HP-IDF (10.35%) > UT-IDF (8.85%). This change can be ascribed to the preferential degradation and fracture of the amorphous regions during the modification process; sharper peak shapes indicate higher crystallinity. Existing literature indicates that the thermal stability of DF increases with increasing crystallinity [42].

#### 3.3.4. Viscosity (VS)

As shown in Figure 4C, the rheological behavior analysis revealed that the four IDF suspensions exhibited typical characteristics of non-Newtonian pseudoplastic fluids, with viscosity decreasing as the shear rate increased, demonstrating a shear-thinning effect [43]. Compared with CB-IDF, all modification methods increased the apparent viscosity of the system, with HP-IDF showing the greatest increase in viscosity, followed by MT-IDF and UT-IDF. This difference was particularly pronounced at low shear rates, whereas at high shear rates, the difference diminished and tended to stabilize. The increase in HP-IDF viscosity can be ascribed to the moderate depolymerization of molecular chains induced by ultra-high-pressure treatment; the increased exposure of hydrophilic groups enhances the interaction between fibers and water molecules, thereby forming a more stable three-dimensional network structure [44]. HP-IDF may therefore have good potential for application as a natural thickener and stabilizer in food systems.

#### 3.3.5. X-Ray Photoelectron Spectroscopy (XPS)

**Figure 5:**
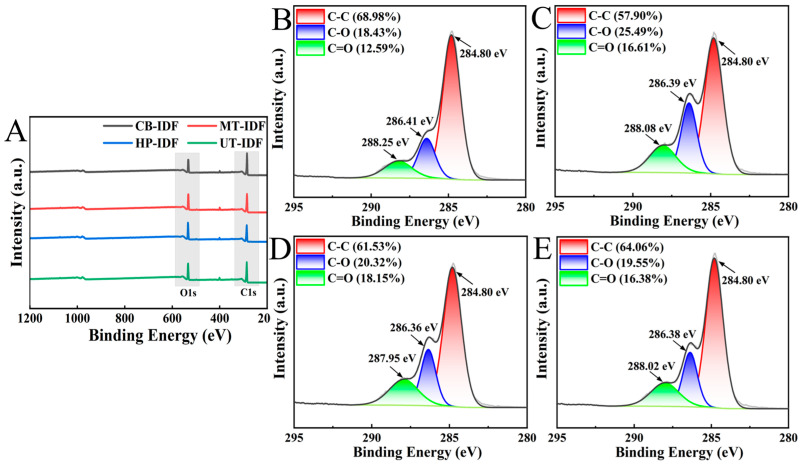
XPS survey spectra of IDF samples subjected to different modification methods (**A**); high-resolution C1s spectra of CB-IDF (**B**); high-resolution C1s spectra of MT-IDF (**C**); high-resolution C1s spectra of HP-IDF (**D**); high-resolution C1s spectra of UT-IDF (**E**).

XPS can effectively identify and distinguish the different chemical forms and bonding states of elements in a sample. The survey spectrum is shown in Figure 5A, where the characteristic peaks of C1s (~284 eV) and O1s (~531 eV) are clearly visible. The C-C, C-O, and C=O chemical bonds within the IDF framework of okara are the primary sources of the C1s spectral peaks, while the C-O bonds and carboxyl groups provide the main signal sources for the O1s spectral peaks [45]. Further peak fitting of the high-resolution C1s spectra revealed that the C1s spectra of all samples exhibited three peaks at approximately 284 eV(C-C), 286 eV(C-O), and 288 eV (C=O) (Figure 5B–E). The proportions of the carbon components in CB-IDF were as follows: C-C bonds (68.98%), C-O bonds (18.43%), and C=O bonds (12.59%). This result is consistent with the chemical structural characteristics of the fiber backbone, in which C-C and C-O bonds are dominant [1]. Compared with CB-IDF, all three modification methods reduced the relative proportion of C-C bonds while increasing the content of C-O and C=O bonds to varying degrees; this may provide indirect evidence for the appearance of the characteristic ester bond peak near 1246 cm^−1^ in the FTIR spectrum. The high-intensity mechanical action generated by physical modification can disrupt the fiber framework and adequately expose internal active groups, thereby altering the microstructure and chemical composition [9]. This restructuring of the surface chemical state further confirms the effective deconstruction of the fiber backbone by the modification methods at the molecular scale.

#### 3.3.6. Thermogravimetric Analysis (TG)

**Figure 6:**
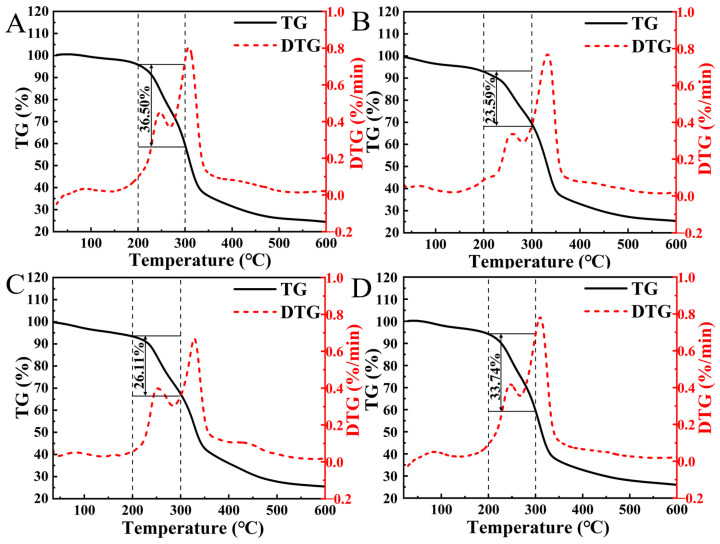
Thermogravimetric curves of IDF samples subjected to different modification methods. CB-IDF (**A**); MT-IDF (**B**); HP-IDF (**C**); UT-IDF (**D**).

High temperatures can cause DF to degrade to some extent, thereby affecting the thermal stability of the material. As shown in Figure 6, the DTG curves in Figure 6A–D primarily exhibit three distinct peaks. In the TG curves, the weight loss in the first stage (25–200 °C) is mainly ascribed to the removal of water bound by hydrophilic groups on the fiber surface [46]. In the second stage (200–300 °C), the mass loss of CB-IDF was 36.50%, while those of MT-IDF, HP-IDF, and UT-IDF were 23.59%, 26.11%, and 33.74%, respectively. Furthermore, the main DTG peak shifted toward higher temperatures, indicating that modification enhanced the thermal stability of the fibers. Among these samples, MT-IDF and HP-IDF exhibited the lowest mass loss. This suggests that the effects of microjet homogenization and ultra-high pressure on the fiber structure promoted the effective degradation of the amorphous regions. These results indicate that the improved thermal stability may stem from an increase in the crystallinity of the samples [47]. This is consistent with the aforementioned XRD results. Physical modification enhanced the thermal resistance of the IDF samples to varying degrees, improving their processing suitability for applications in high-temperature food systems.

### 3.4. Physical and Chemical Properties Analysis

#### 3.4.1. Hydration Characteristics Analysis

**Figure 7:**
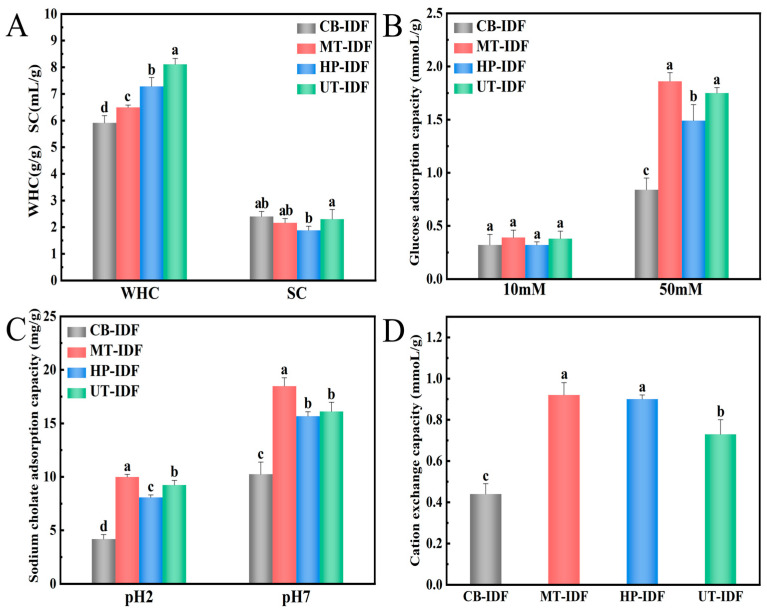
Hydration properties (WHC and SC) (**A**); glucose adsorption capacity (GAC) (**B**); sodium cholate adsorption capacity (SCAC) (**C**); cation exchange capacity (CEC) (**D**). CB-IDF: unmodified IDF; MT-IDF: IDF modified by microjet homogenization; HP-IDF: IDF modified by ultra-high pressure; UT-IDF: IDF modified by ultrasound. Different letters indicate statistically significant differences between groups (*p* < 0.05).

Figure 7A shows the changes in water-holding capacity (WHC) and swelling capacity (SC) of IDF. In contrast to CB-IDF, the WHC of all three modified samples was markedly elevated (*p* < 0.05), with UT-IDF showing the greatest enhancement: 1.11 times that of HP-IDF, 1.25 times that of MT-IDF, and 1.37 times that of CB-IDF. This may be due to ultrasonic modification, which generates tiny pores or voids within the IDF granules, thereby increasing the number of binding sites for water and enhancing their hydrophilicity [48]. Regarding the SC results, UT-IDF was significantly higher than HP-IDF, while showing no remarkable variation compared with CB-IDF and MT-IDF. Previous studies indicate that modification treatment can increase the surface porosity of the fibers; however, the simultaneous degradation of cellulose and hemicellulose may have an offsetting influence on the improvement of water-holding capacity [49].

#### 3.4.2. Glucose Adsorption Capacity Analysis

GAC serves as a core index for assessing the capacity of dietary fiber to regulate postprandial blood glucose levels. As illustrated in Figure 7B, the GAC of different modified IDF samples exhibited significant differences at glucose concentrations of 10 mM and 50 mM. At 10 mM, there were no notable differences among the groups (*p* > 0.05). However, at a concentration of 50 mM, the adsorption capacity increased significantly, and all modified groups showed significantly higher values than CB-IDF. Intense mechanical action can efficiently disrupt the compact structure of IDF, exposing more pores and internal surface area, thereby providing abundant adsorption sites for glucose molecules to form hydrogen bonds [50,51]. All three physical modification methods effectively enhanced the GAC of IDF, providing a solid theoretical basis for the development of functional products with potential blood-glucose regulating effects.

#### 3.4.3. Sodium Cholate Adsorption Capacity Analysis

IDF with high SCAC can inhibit cholesterol absorption and thereby regulate cholesterol levels [52]. As revealed in Figure 7C, the adsorption capacity of all okara IDF samples was distinctly greater at pH 7 than at pH 2. This may be because bile salts exist in ionic form under acidic conditions, resulting in weaker binding to fiber. In contrast, under neutral conditions, they exist in non-ionic form, allowing them to interact efficiently with both the hydrophilic and hydrophobic sites of the fiber [53]. The adsorption capacity followed the order: MT-IDF (pH 2.0: 9.98 mg/g, pH 7.0: 18.47 mg/g) > UT-IDF (pH 2.0: 9.23 mg/g, pH 7.0: 16.11 mg/g) > HP-IDF (pH 2.0: 8.07 mg/g, pH 7.0: 15.67 mg/g) > CB-IDF (pH 2.0: 4.18 mg/g, pH 7.0: 10.24 mg/g). This may be ascribed to changes in the particle size, microscopic structure, and functional groups of the modified okara IDF.

#### 3.4.4. Cation Exchange Capacity Analysis

The CEC results are shown in Figure 7D. Compared with CB-IDF (0.44 mmol/g), all three modification methods significantly increased the CEC values of the samples, specifically: UT-IDF (0.73 mmol/g), HP-IDF (0.90 mmol/g) and MT-IDF (0.92 mmol/g). Among them, HP-IDF and MT-IDF exhibited the highest CEC values, with no statistically significant difference between them (*p* > 0.05). Following modification, the specific surface area of the fibers increased, thereby exposing more side-chain groups involved in cation exchange, which in turn enhanced the adsorption and exchange capacity for sodium ions in solution [54]. These findings reveal that structural optimization can substantially improve the binding capacity of dietary fiber for ions.

### 3.5. Biological Activity Analysis

#### 3.5.1. *In Vitro* Digestion Rate Analysis

**Figure 8:**
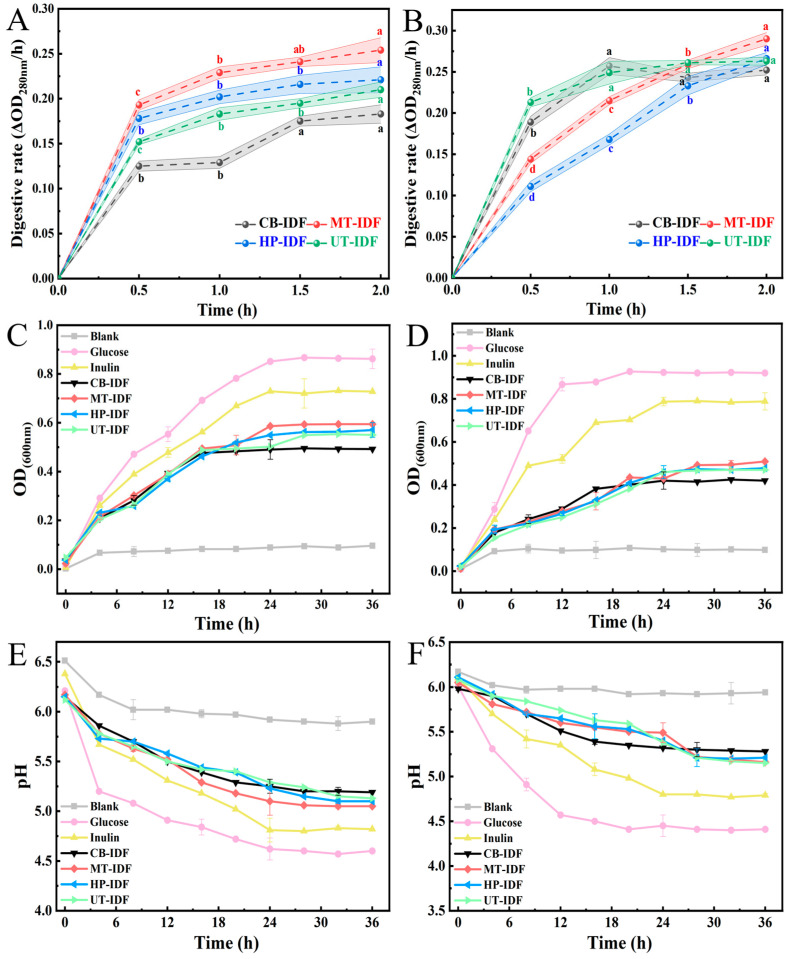
Digestion rate during the gastric phase (**A**); digestion rate during the intestinal phase (**B**); growth curve of *L. acidophilus* (**C**); growth curve of *B. longum* (**D**); pH change in *L. acidophilus* (**E**); pH change in *B. longum* (**F**). Different letters indicate statistically significant differences between groups (*p* < 0.05).

An in vitro simulated digestion rate test was used to evaluate the degradation degree of IDF from okara subjected to different physical modifications in a human gastrointestinal simulation system. During the gastric digestion phase (Figure 8A), the digestion rates of all samples gradually increased over time, and the digestion rates of the modified groups were all significantly higher than that of CB-IDF. Among these, MT-IDF exhibited the highest digestion rate, followed by HP-IDF and UT-IDF, indicating that physical modification can significantly enhance the degradation efficiency of fiber in the gastric environment. During the intestinal digestion phase (Figure 8B), the digestion rates of CB-IDF and UT-IDF increased rapidly within 0.5 h and were higher than those of MT-IDF and HP-IDF until 1.5 h. Subsequently, the digestion rate of MT-IDF peaked at 2 h. This may be ascribed to the fiber’s porous structure and the reconfiguration of surface hydrophilic groups, which provided more sustained binding sites for digestive enzymes [49]. This section only evaluates the fiber degradation ability through in vitro simulation of the digestive system, and cannot fully simulate the physiological environment of the intestinal tract in vivo. Further animal studies are required to verify the digestive performance of modified okara IDF under in vivo conditions.

#### 3.5.2. In Vitro Probiotic Activity Analysis

*L. acidophilus* and *B. longum* have received widespread attention owing to their key roles in improving dysbiosis. This study evaluated the prebiotic efficacy of okara IDF toward *L. acidophilus* and *B. longum* in a culture system without additional carbon sources. Findings revealed that growth in the glucose and inulin groups was significantly greater than that in the other groups. However, although the okara IDF groups showed lower growth than the glucose and inulin groups, they were all significantly greater than the blank control group, confirming that okara IDF can be selectively fermented and utilized by intestinal probiotics and possesses potential prebiotic activity. The modified groups performed better than the CB-IDF group, which may be closely related to their structural characteristics. For *L. acidophilus* (Figure 8C,E), UT-IDF and MT-IDF exhibited the highest OD values at 24–36 h, showing trends highly consistent with pH changes and a smaller gap compared with the inulin group. It is possible that the consumption of carbon substrates facilitated probiotic proliferation and further reduced the pH of the culture medium [29]. For *B. longum* (Figure 8D,F), growth in the MT-IDF, HP-IDF, and UT-IDF groups leveled off after 20 h, with probiotic activity being weaker than that of the glucose and inulin groups. The pH decrease in the IDF systems differed significantly between the two probiotic strains. This may be related to differences in the carbon source utilization pathways among different strains [55]. Relevant research demonstrates that dietary fiber, after resisting hydrolysis by digestive enzymes, is fermented and utilized by the gut microbiota, thereby promoting the proliferation of beneficial bacteria [56,57]. In summary, okara IDF can serve as a promising substrate for the development of prebiotic-related products. This section used only in vitro culture systems to evaluate the probiotic potential of okara IDF and therefore lacks verification in the complex symbiotic environment of gut microbiota. Subsequent in vivo animal experiments should be conducted to address the limitations of in vitro models.

## 4. Discussion

This study systematically compared the differential regulatory effects of three physical modification methods MT, HP, and UT on the compositional profiles, microstructure, physicochemical properties and functional characteristics of IDF extracted from okara. Distinct action mechanisms of the three treatments lead to divergent structural and functional performance of modified fiber.

Basic Compositions:

All three physical modification techniques disintegrate dense okara fiber aggregates via mechanical force and eliminate impurities embedded within the polysaccharide backbone. The high-intensity shear generated during microjet homogenization breaks down fiber aggregates more thoroughly, delivering fiber with superior purity. This compositional variation trend aligns with the findings documented by Tian et al. [19]. In terms of IDF extraction yield, ultrasound induces instantaneous shockwaves that rupture okara cell walls and liberate bound IDF trapped in cell wall matrices, which accounts for its maximum extraction efficiency. This underlying mechanism is corroborated by the research outcomes of Yu et al. [30]. HP treatment triggers moderate depolymerization of fiber molecular chains through omnidirectional pressure infiltration, which effectively optimizes the hydration performance of dietary fiber, consistent with the trends reported by Zuo et al. [16]. Physical modification features eco-friendly performance and great feasibility for industrial scale-up.

Physicochemical Properties:

Distinct energy transfer patterns of the three modification approaches lead to differentiated microstructure and physicochemical performance of okara IDF. MT can fully break large-sized fiber particles and form a loose and porous surface structure, which is in accordance with the findings of Li et al. [14]. HP modification only relaxes the cross-linked molecular network of fibers, resulting in limited fiber micronization and slight improvement in the physicochemical properties of the obtained samples; this variation trend is similar to the reports of Ma et al. [20]. UT disassembles fiber aggregates merely through surface cavitation etching and cannot deeply destroy the ordered crystalline zones inside fibers. Previous studies by Hussain et al. [18] and Shi et al. [21] only emphasized the merit of ultrasound in enhancing hydration properties, without in-depth discussion of its disadvantages. This study clarifies the properties of UT-IDF featuring excellent hydration capacity yet insufficient thermal stability, which provides more comprehensive insights compared with the existing one-dimensional research conclusions.

In Vitro Functional Properties:

Improving the functional properties of okara-based DF is critical to boosting consumer acceptability [58]. Benefiting from its porous surface and high proportions of C–O and C=O bonds, MT-IDF provides abundant adsorption sites and exhibits superior adsorption activity compared with HP-IDF and UT-IDF. This trend is consistent with the theory proposed by Tang et al. [11] that particle refinement exerts a positive regulatory effect on adsorption performance. Results of in vitro digestion and probiotic activity assays reveal that the porous fiber structure offers continuous binding sites for digestive enzymes, leading to milder and longer enzymatic degradation. Modified DF can sustainably release fermentable carbon sources, thereby possessing stronger probiotic activity than unmodified fiber, which agrees with the findings of Yu et al. [28]. In this study, two typical intestinal probiotics, *Lactobacillus acidophilus* and *Bifidobacterium longum*, were selected. The proliferation dynamics of strains were simultaneously monitored at multiple time gradients, which enables a more objective and comprehensive elucidation of the structure–activity relationship between the microstructure of modified fiber and its prebiotic potential.

Future Perspectives:

Future research should focus on in vivo digestion, metabolism, and gut microbiota interactions to elucidate the specific mechanisms by which modified okara IDF exerts its functional effects. Additional studies utilizing real food matrices are required to investigate critical indicators including sensory quality, storage stability and processing scalability, so as to further validate its practical performance and industrialization potential.

## 5. Conclusions

This study verified that MT, HP, and UT can all improve the quality of okara IDF. Nevertheless, their modification mechanisms differ in structural regulation, functional characteristics, and application suitability. According to the results of the present study, MT-IDF exhibited excellent particle refinement (D90: 91.51 μm) and a larger specific surface area (0.33 m^2^/g) and a porous structure, as well as superior adsorption performance (CAC (pH 2; pH 7): 9.98 mg/g; 19.47 mg/g, SCAC (50 mM): 1.86 mmol/g, CEC: 0.92 mmol/g), thermal stability, and prebiotic activity (*Lactobacillus acidophilus* 36h-OD_600_: 0.594; *Bifidobacterium longum* 36h-OD_600_: 0.509), suggesting that it may be more suitable for high-value applications such as functional foods and prebiotic formulations. HP-IDF showed a significant increase in viscosity and may be more suitable as a natural thickener in the food system. However, its particle refinement was less effective than that of MT-IDF and UT-IDF. UT-IDF, conversely, demonstrated outstanding hydration capacity (WHC: 8.11 g/g, SC: 2.30 mL/g) and may have greater potential in high-moisture food systems. In summary, physical modification can effectively improve the resource utilisation rate of okara.

## Figures and Tables

**Table 1:** Changes in color and particle size of IDF samples subjected to different modification methods.

	CB-IDF	MT-IDF	HP-IDF	UT-IDF
Color parameters				
L*	62.87 ± 0.32 ^c^	74.20 ± 0.51 ^a^	71.09 ± 0.91 ^b^	70.97 ± 0.49 ^b^
a*	5.77 ± 0.03 ^d^	6.89 ± 0.06 ^a^	6.15 ± 0.05 ^c^	6.67 ± 0.08 ^b^
b*	6.02 ± 0.16 ^c^	11.00 ± 0.49 ^b^	11.43 ± 0.07 ^b^	15.44 ± 0.14 ^a^
Particle size (μm)				
D10	14.38 ± 0.35 ^a^	7.44 ± 0.23 ^d^	8.88 ± 0.30 ^b^	8.22 ± 0.19 ^c^
D50	64.30 ± 0.62 ^a^	38.20 ± 1.14 ^c^	49.22 ± 1.15 ^b^	39.77 ± 2.06 ^c^
D90	175.04 ± 1.73 ^a^	91.51 ± 1.50 ^d^	108.73 ± 4.37 ^b^	101.07 ± 2.01 ^c^
D [3,2]	33.26 ± 0.30 ^a^	18.01 ± 0.88 ^c^	22.41 ± 2.06 ^b^	19.49 ± 0.50 ^c^
D [4,3]	81.55 ± 0.65 ^a^	44.67 ± 0.68 ^d^	55.64 ± 2.49 ^b^	48.34 ± 1.21 ^c^
SSA (m^2^/g)	0.18 ± 0.00 ^d^	0.33 ± 0.00 ^a^	0.27 ± 0.00 ^c^	0.31 ± 0.00 ^b^

Different letters within the same row denote statistically significant differences among groups (*p* < 0.05). SSA: specific surface area.

## Data Availability

The original contributions presented in this study are included in the article. Further inquiries can be directed to the corresponding author.
